# Epidemiology of COVID-19 in Northern Ireland, 26 February 2020–26 April 2020

**DOI:** 10.1017/S0950268821000224

**Published:** 2021-01-29

**Authors:** J. Pett, P. McAleavey, P. McGurnaghan, R. Spiers, M. O'Doherty, L. Patterson, J. Johnston

**Affiliations:** 1 Public Health Agency Northern Ireland, Belfast, Northern Ireland; 2 UK Field Epidemiology Training Programme, Global Public Health Division, Public Health England, London, UK

**Keywords:** Coronavirus, COVID-19, pandemic, surveillance, transmission

## Abstract

This paper describes the epidemiology of coronavirus disease 2019 (COVID-19) in Northern Ireland (NI) between 26 February 2020 and 26 April 2020, and analyses enhanced surveillance and contact tracing data collected between 26 February 2020 and 13 March 2020 to estimate secondary attack rates (SAR) and relative risk of infection among different categories of contacts of individuals with laboratory confirmed severe acute respiratory syndrome-coronavirus-2 (SARS-CoV-2) infection. Our results show that during the study period COVID-19 cumulative incidence and mortality was lower in NI than the rest of the UK. Incidence and mortality were also lower than in the Republic of Ireland (ROI), although these observed differences are difficult to interpret given considerable differences in testing and surveillance between the two nations. SAR among household contacts was 15.9% (95% CI 6.6%–30.1%), over 6 times higher than the SAR among ‘high-risk’ contacts at 2.5% (95% CI 0.9%–5.4%). The results from logistic regression analysis of testing data on contacts of laboratory-confirmed cases show that household contacts had 11.0 times higher odds (aOR: 11.0, 95% CI 1.7–70.03, *P*-value: 0.011) of testing positive for SARS-CoV-2 compared to other categories of contacts. These results demonstrate the importance of the household as a locus of SARS-CoV-2 transmission, and the urgency of identifying effective interventions to reduce household transmission.

## Introduction

On 31 December 2019, a cluster of cases of pneumonia of unknown origin was reported to the World Health Organisation (WHO) by Chinese authorities in Wuhan [1]. These are now known to have been cases of coronavirus disease 2019 (COVID-19), caused by the virus severe acute respiratory syndrome-coronavirus-2 (SARS-CoV-2), the early epidemiology and characteristics of which are described extensively elsewhere [2–10].

The first suspected COVID-19 case in Northern Ireland (NI) was reported to the Public Health Agency (PHA) and tested on 22 January 2020. The first laboratory-confirmed case reported in the UK occurred in England on 29 January 2020. The first laboratory-confirmed case in NI was reported on 26 February 2020, and on 29 February 2020, COVID-19 was added to the schedule of notifiable diseases in NI [11].

This paper describes the epidemiology of COVID-19 in NI between 26 February 2020 and 26 April 2020. It also describes enhanced surveillance data on the first 39 cases reported in NI, for whom enhanced surveillance data were available and contact tracing and monitoring was carried out.

## Methods

### Case definitions

The UK has used five different case definitions for COVID-19 since the pandemic began, and these are presented in Appendix 1.

### Contact definitions

Contacts of cases are categorised as household, high and low risk (Appendix 2). During the study period, contacts were only offered testing if they reported symptoms included in the case definition in use at the time.

### SARS-CoV-2 laboratory testing

Testing was made available from PHE's National Reference Laboratory for all of the UK on 24 January 2020. On 7 February 2020, SARS-CoV-2 the Regional Virus Laboratory, Belfast Health and Social Care (HSC) Trust began conducting testing locally. Testing was rolled out to the other four HSC Trust labs between 23 March 2020 and 13 May 2020.

Between 22 January 2020 and 13 March 2020, testing was performed for all individuals meeting the case definition in use at the time testing was carried out. On 13 March 2020, testing was expanded to HSC workers and members of their household who meet the suspected case definition but do not require hospitalisation. Testing was expanded again on 4 April 2020 to include other ‘key workers’ (see Appendix 3 for list of key worker categories) who meet the suspected case definition but do not require hospitalisation.

### Data sources

#### Routine virology data

The PHA Health Protection Directorate laboratory surveillance system collates SARS-CoV-2 laboratory data on all tests from HSC Trust laboratories. Laboratory data are then collated to enable monitoring of individuals rather than tests performed by laboratories using the Organism-Patient-Illness-Episode (OPIE) principle [12].

Data on all individuals with a positive test result between 26 February 2020 and 26 April 2020 were extracted from the PHA Health Protection laboratory surveillance system. These data also include date of birth, sex and residential postcode for each case.

#### Enhanced surveillance data

The PHA Health Protection surveillance team developed a bespoke electronic-enhanced surveillance system using web-based forms for collecting surveillance information on cases and contacts, aligned with PHE protocol adapted from WHO guidance [13]. Contacts were identified via phone calls with the index case, or a contact of the index case where the index case was unable to provide these data.

Enhanced surveillance and contacts data for the 39 cases reported in NI during the initial period of contact tracing were extracted from this dataset, including demographics, travel history, exposures, occupation, symptoms and outcomes (see Appendix 4). Contact data include demographics and categorisation into household, high risk or low risk (Appendix 2). During the initial phone call with contacts of index cases, contacts were asked if they were symptomatic and if they reported symptoms testing was arranged. Where contacts reported no symptoms, they were advised to contact PHA to arrange testing if they experienced symptoms over the 14 days since their last contact with the index case. Where data were missing from the enhanced surveillance datasets, individual case records from HPZone, an electronic case-management system in use throughout the UK, were reviewed to complete missing fields wherever possible.

### Data analysis

We described COVID-19 cases from the routine virology surveillance dataset by age, sex, local government district (LGD), and deprivation quintile. We also described COVID-19 cases from the enhanced case and contact surveillance datasets by age, sex, exposures, symptoms and outcome. We estimated secondary attack rates (SAR) and secondary clinical attack rates in household, high-risk and low-risk contacts and by age group. We then estimated incubation periods and serial intervals using symptom onset dates in transmission pairs where data were available. Where the secondary case's reported date of symptom onset was the same as the date of most recent contact with the primary case, one day was assigned as the minimum incubation period.

We then carried out multivariate logistic regression analysis to estimate the association between contact risk category, age and sex and testing positive for SARS-CoV-2. We calculated crude and adjusted odds ratios, and 95% credible intervals (CI).

## Results

### Descriptive epidemiology in NI

**Fig. 1.**
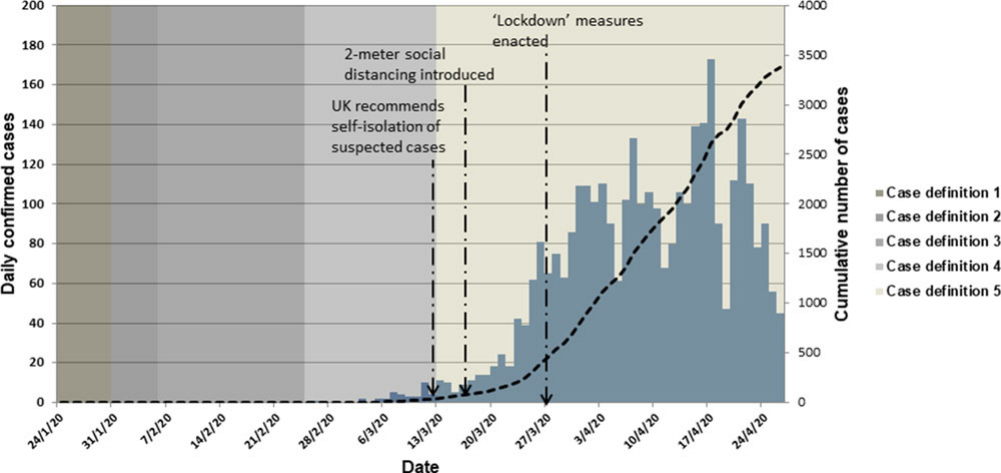
Daily number of confirmed COVID-19 cases in NI, by reporting date, 24 January 2020 to 26 April 2020 (*n* = 3380).

In all, 3380 individuals tested positive for SARS-CoV-2 in NI during the study period (Fig. 1) giving NI a cumulative incidence per 100 000 population of 179.6 (95% CI 173.6–185.8). This is lower than cumulative incidence in England at 208.4 (95% CI 207.2–209.6) per 100 000 population [14], and below Wales at 308.7 (95% CI 302.6–314.9) [15], and Scotland at 189.3 (95% CI 185.7–193.0) [16] over this period.

This is also lower than the Republic of Ireland (ROI), where cumulative incidence was 392.8 (95% CI 387.3–398.4) per 100 000 population [17]. This is likely explained by differences in testing and contact tracing policy between NI and the ROI, and these different approaches to surveillance and policy make interpretation of total case numbers difficult to interpret.

### Crude COVID-19 mortality rate

In total, 334 individuals with laboratory-confirmed SARS-CoV-2 infection died in NI over the study period, giving a crude COVID-19 mortality rate of 17.7 per 100 000 population (95% CI 15.9–19.8). This is lower than the crude COVID-19 mortality rates per 100 000 population in England, Scotland and Wales, which are 38.9 (95% CI 38.3–39.4), 22.9 (95% CI 21.6–24.2) and 28.6 (95% CI 26.8–30.5), respectively. It is also lower than the crude mortality rate in the ROI of 20.6 per 100 000 population (95% CI 19.3–21.9), although the 95% CIs for NI and the ROI overlap slightly.

### Age and sex distribution

**Fig. 2.**
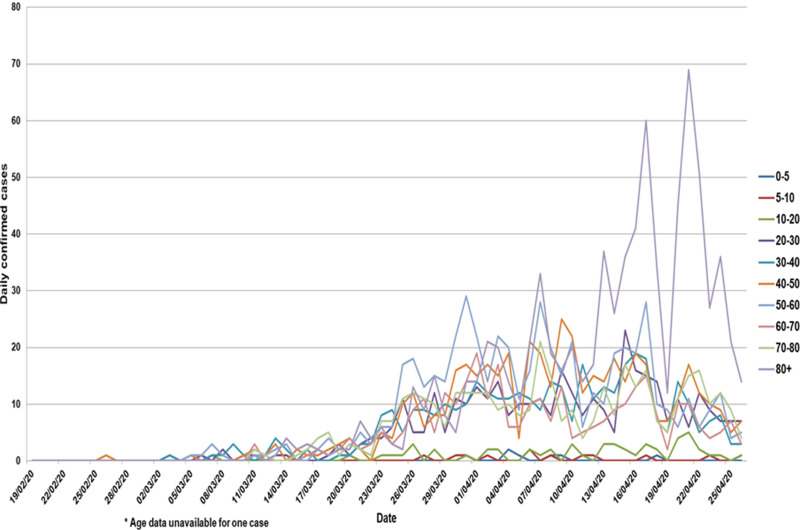
Daily COVID-19 cases by age group and specimen date, NI, 19 February 2020 to 26 April 2020 (*n* = 3379)*.

**Fig. 3.**
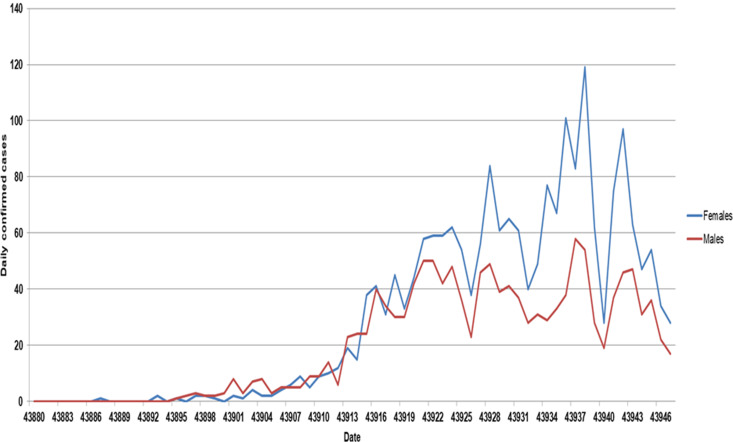
Daily COVID-19 cases by sex and specimen date, NI, 19 February 2020 to 26 April 2020 (*n* = 3380).

Cases ranged in age from <1 month to 104 years old (median: 57.1, IQR: 40.5, 80.3). In all, 61.9% (2093) were aged 50 or over and 59.8% (2022) were female individuals (Table 1). The age and sex profile of reported cases in NI has changed over time (Figs 2 and 3), with the number of cases that are female and in older age groups increasing relative to males and younger age groups. Cases were reasonably evenly distributed between deprivation quintiles.

**Table 1.** Demographic characteristics of confirmed COVID-19 cases in NI, 26 February 2020 to 26 April 2020, *n* = 3380

Demographics	*n*	%
Female	2022	59.8
Age range (years)	0–104	
Median age	57.1	
Age group	***n***	**%**
<5	8	0.2
5–10	11	0.3
10–20	54	1.6
20–30	372	11.0
30–40	376	11.1
40–50	466	13.8
50–60	540	16.0
60–80	697	20.6
80 +	856	25.3
LGD	***n***	**%**
Belfast	1058	31.3
Armagh, Banbridge and Craigavon	398	11.8
Lisburn and Castlereagh	341	10.1
Antrim and Newtonabbey	306	9.1
Mid and East Antrim	234	6.9
Ards and North Down	210	6.2
Newry, Mourne and Down	201	5.9
Causeway Coast and Glens	185	5.5
Derry and Strabane	172	5.1
Mid Ulster	166	4.9
Fermanagh and Omagh	68	2.0
Residence outside NI or unknown	41	1.2
Deprivation quintile	***n***	**%**
1 (least deprived)	701	20.7
2	601	17.8
3	747	22.1
4	723	21.4
5 (most deprived)	825	24.4
Unknown	41	1.2

### Descriptive epidemiology of enhanced surveillance and contact tracing data

**Fig. 4.**
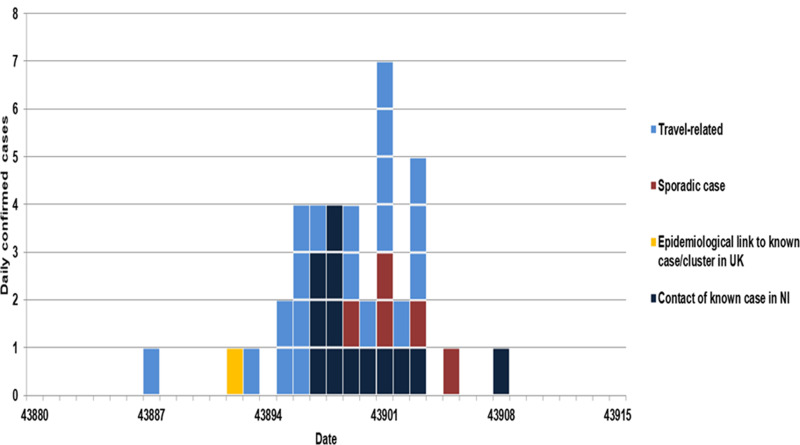
COVID-19 cases identified through enhanced surveillance and contact tracing by specimen date and exposure, NI, 19 February 2020 to 25 March 2020 (*n* = 39).

Between 26 February 2020 and 7 March 2020, all cases [10] were associated with travel to an affected area that was included in the UK case definition (Fig. 4). Seven out of the eight cases between 7 March 2020 and 9 March 2020 were part of two clusters linked to one primary case who tested positive on 6 March 2020.

On 9 March 2020 the first sporadic case (a case without a relevant travel history or known epidemiological link to a case or cluster in NI or elsewhere in the UK occurred) tested positive. A further four sporadic cases occurred between 11 March 2020 and 15 March 2020.

### Exposures

**Fig. 5.**
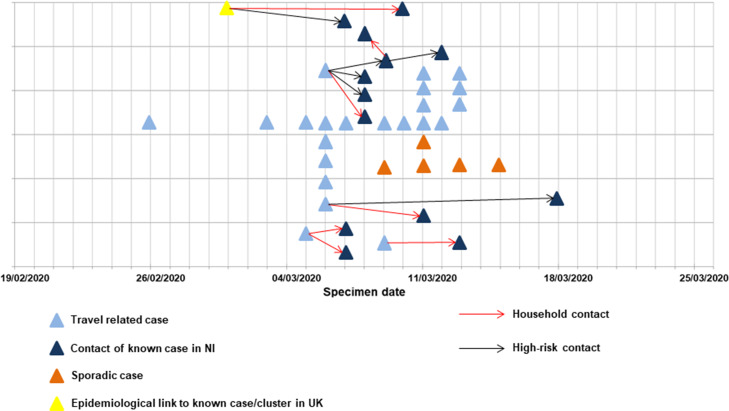
COVID-19 cases identified through enhanced surveillance and contact tracing, by specimen date, exposure, and transmission setting, NI, 19 February 2020 to 25 March 2020 (*n* = 39).

Between 26 February 2020 and 13 March 2020, 51.3% [18] of all cases travelled to a known affected area outside the UK in the 14 days before notification, of whom 10 (50.0%), seven (35.0%) and two (10.0%) travelled to Italy, Austria and France, respectively. One (5.0%) individual had recently returned from a cruise. In all, 48.7% [19] reported contact with an individual or individuals with COVID-19-like symptoms in the previous 14 days, and 33.3% [13] were contacts of known cases in NI (Fig. 5). A percentage of 7.7 [3] were employed as healthcare workers (HCW) at the time of reporting, two of whom were primary cases and one of whom was a secondary case (Table 2).

**Table 2.** Exposures for COVID-19 cases identified through enhanced surveillance and contact tracing 26 February 2020 to 13 March 2020, *n* = 39

Exposures	*n*	%
Reported contact with individual(s) with COVID-19-like symptoms in previous 14 days		
Yes	19	48.7
No	14	35.9
Unknown	6	15.4
Travel to affected area outside UK	20	51.3
Italy	10	50.0
Austria	7	35.0
France	2	10.0
Cruise ship	1	5.0
Contact of known case in NI	13	33.3
Contact of known case/cluster in UK	1	2.6
Sporadic case (no reported exposures)	5	12.8
Occupation		
HCW	3	7.7
Pilot	1	2.6
Retail manager	1	2.6
Unknown	34	87.2

### Age−sex distribution

Cases identified through enhanced surveillance ranged in age from 13 to 86 years old, with the majority (64.1%) aged below 50 years (Table 3); 59.0% [20] of the cases were male. The age and sex distribution of these early cases differs from those reported after 13 March 2020, and this is likely due to changes to the UK case definition and testing policy which meant the majority of cases reported to PHA were hospitalised patients, individuals living in care homes, and to a lesser extent, healthcare and other key workers.

**Table 3.** Demographic characteristics of COVID-19 cases identified through enhanced surveillance and contact tracing, NI, 26 February 2020 to 18 March 2020, *n* = 39

Demographics	*n*	%
Male	23	59.0
Age range	13–86	
Median age	39	
Age group		
<5	0	0.0
5–10	0	0.0
10–20	1	2.3
20–30	7	17.9
30–40	10	25.6
40–50	7	17.9
50–60	7	17.9
60–70	2	5.1
70–80	3	7.6
80 +	2	5.1

### Symptoms and co-morbidities

Data on reported symptoms and co-morbidities were available for 36 (92.3%) cases. Of these, 91.7% [21] reported at least one symptom at the time of, or in the 7 days prior to, notification. Of these, 69.4% [22] reported fever, 66.6% [23] reported a cough and 22.2% [8] reported shortness of breath. The least commonly reported symptoms were delirium (2.9%), myalgia (2.9%) and fatigue (2.9%). Three (8.3%) cases reported experiencing no symptoms (Table 4).

**Table 4.** Clinical features of COVID-19 cases identified through enhanced surveillance and contact tracing, NI, 26 February 2020 to 18 March 2020, *n* = 39

Clinical features	*N*
Symptoms (*n* = 36)	
Fever	25/36
Cough	24/36
Shortness of breath	8/36
Chest pain	3/36
Headache	3/36
Fatigue	2/36
Coryzal	2/36
Myalgia	1/36
Delirium	1/36
No reported symptoms	3/36
Co-morbidities (*n* = 36)	
Asthma	2/36
Hypertension	2/36
Tuberculosis	1/36
No reported co-morbidities	31/36
Admitted to hospital (*n* = 35)	
Yes	11/35
Outcome (*n* = 39)	
Died	1/39
Unknown	34/39

### Other outcomes

Data on hospital admission and mortality during the period 26 February 2020 to 13 March 2020 were available for 35 (89.7%) and 39 (100.0%) cases, respectively. Of these, 31.4% [11] were admitted to hospital, and one case (2.9%) died.

### Analytical epidemiology of enhanced surveillance and contact tracing data

Complete information on contacts of cases was available for 27 cases (69.2%). Ten (25.6%) cases were identified after the suspension of contact tracing on 13 March 2020, and so contact tracing data were not available for these individuals. Five (50.0%) of these had a positive test result on or after 13 March 2020, and five (50.0%) were contacts of confirmed cases and had a positive test result within 7 days of 13 March 200, which were able to identify through linking contact tracing data to virology data. The specimen date for the last of these cases was 18 March 2020. For the two (5.1%) remaining cases for whom no contact tracing data were available, we were unable to determine whether this was because they reported having no household, high- or low-risk contacts, or if these data were simply not collected.

Totally, 392 contacts were identified from the 27 cases for whom contact tracing data were available, with the median number of contacts per case was 4 (min: 1.0, max: 57.0, IQR: 2.5, 17.5). Overall, 44 (11.5%), 238 (60.7%) and 110 (28.1%) were household, high- and low-risk contacts, respectively. Of the 392 contacts, 44 (11.2%) were tested for SARS-CoV-2, of which 13 (29.5%) were positive, one (2.3%) initially tested negative before testing positive 12 days later and the remaining 30 (68.2%) were negative.

### Transmission dynamics

Between 26 February 2020 and 18 March 2020, 13 (33.3%) cases were contacts of previously reported cases within NI. Seven (53.8%) were household contacts, six (46.2%) were high-risk contacts and there were no cases reported among low-risk contacts.

SAR and clinical attack rates for different categories of contact and by age group are shown in Table 5. We estimate that the SAR among household contacts (15.9%, 95% CI 6.6%–30.1%) is over 6 times higher than among high-risk contacts (2.5%, 95% CI 0.9%–5.4%). Six (46.2%) secondary cases' earliest reported contact with the primary case was before the primary case's reported date of symptom onset, of which two secondary cases' (33.3%) only reported contact with the primary case was before the primary case's reported onset of symptoms, indicating possible pre-symptomatic transmission.

**Table 5.** Secondary and clinical attack rates among COVID-19 cases identified through enhanced surveillance and contact tracing, NI, 26 February 2020 to 18 March 2020, *n* = 392

	Number of cases	Number of contacts	Proportion of contacts tested (%)	Attack rate (%)	Clinical attack rate (%)
Risk category					
Household	7	44	15.9	15.9 (6.6–30.1)	15.9 (6.6–30.1)
High risk	6	238	11.7	2.5 (0.9–5.4)	1.7 (0.5–4.2)
Low risk	0	110	3.6	0.0	0.0
Age group (*n* = 259)^a^					
<5	0	8	0.0	0.0	0.0
5–10	0	5	0.0	0.0	0.0
10–20	1	12	16.7	8.3 (0.2–38.5)	8.3 (0.2–38.5)
20–30	4	57	19.3	7.0 (1.9–17.0)	3.5 (0.4–12.1)
30–40	4	55	10.9	7.3 (2.0–17.6)	7.3 (2.0–17.6)
40–50	2	50	12.0	4.0 (0.5–13.7)	4.0 (0.5–13.7)
50–60	1	30	6.7	3.3 (0.1–17.2)	3.3 (0.1–17.2)
60–70	0	26	3.8	0.0	0.0
70–80	1	10	10.0	10.0 (0.3–44.5)	10.0 (0.3–44.5)
80 +	0	5	0.0	0.0	0.0

a Age data not available for 135 (34.3%) contacts.

Age data were available for 259 (65.7%) contacts. By age group, the highest attack rate observed was in 20–30-year olds at 19.3%. There were no cases reported in under 5-year, 5–10-year olds, 60–70-year olds and contacts aged 80 and older. The clinical attack rate was highest in 70–80-year olds at 10.0% (95% CI 0.3–44.5%). The 95% CIs for all age groups overlap, however, and given the small number of secondary cases in our study we were unlikely to detect evidence of variation in attack rates by age group.

Six clusters produced the 13 secondary cases identified in NI. The number of secondary cases in these clusters ranged from one to four, with a median cluster size of two. The median minimum incubation period in the six clusters was 2 days (range: 1–12) (Table 6), the median maximum incubation period was 6 (range: 3–16), and the median serial interval was 6 (range: 2–13).

**Table 6.** Incubation periods and serial intervals for cases in six COVID-19 clusters identified in NI, 26 February 2020 to 18 March 2020, *n* = 13

	Minimum incubation period (days)	Maximum incubation period (days)	Serial interval (days)
Cluster A			
Secondary case 1	1	5	5
Secondary case 2^a^	NA	NA	NA
Secondary case 3^a^	NA	NA	NA
Secondary case 4	3	4	6
Cluster B			
Secondary case 1	1	7	7
Secondary case 2	1	6	6
Cluster C			
Secondary case 1	1	5	2
Secondary case 2	3	3	2
Cluster D			
Secondary case 1	9	12	9
Secondary case 2	5	5	3
Cluster E			
Secondary case 1	1	6	3
Secondary case 2	12	16	13
Cluster F			
Secondary case 1	2	9	8

a Case reported no symptoms.

To further investigate infection risk among different categories of contacts, we performed a multivariate logistic regression comparing test positivity in household contacts of confirmed cases who were tested for SARS-CoV-2 compared to high- and low-risk contacts. Analysis of data on the 44 contacts tested shows that, after adjusting for age and sex, household contacts had 11.0 (95% CI 1.7–70.3, *P* = 0.011) times higher odds of testing positive for SARS-CoV-2 compared to high- and low-risk contacts (Table 7). We found no evidence of association between age (OR: 1.0, 95% CI 0.9–1.1, *P* = 0.884) or sex (OR: 0.5, 95% CI 0.1–2.9, *P* = 0.462) and testing positive for SARS-CoV-2.

**Table 7.** Logistic regression analysis of association between contact risk category, age and sex and testing positive for SARS-CoV-2, *n* = 44

	**Crude odds ratio (95% CI)**	***P*-value**	**Adjusted odds ratio (95% CI)**	***P*-value**
Risk category				
High- and low-risk contacts	1.0			
Household	**16.9** (**2.8–102.4)**	**0**.**002**	**11.0** (**1.7–70.3)**	**0**.**011**
Age	1.0 (0.9–1.1)	0.770	1.0 (0.9–1.1)	0.884
Sex				
Female	1.0			
Male	0.4 (0.1–1.5)	0.185	0.5 (0.1–2.9)	0.462

Note: The significance of bold is to highlight that only being a household contact showed statistical evidence of being associated with higher odds of testing positive.

## Limitations

This paper has at least six limitations.

First, the enhanced surveillance and contact tracing data cover the early stages of the COVID-19 epidemic in NI, and largely represents the epidemiology of imported cases (53.9% of cases acquired their infection outside of NI) and their contacts. It is therefore potentially less representative of the period of sustained community transmission that followed introduction of SARS-CoV-2 in NI [24].

Second, as identification of contacts was dependent on self-reporting by index cases, household contacts are likely overrepresented in our sample which may bias our results.

Third, cases reported after the suspension of contact tracing on 13 March 2020 represent the epidemiology of hospitalised cases only, and to a lesser extent, healthcare and other key workers that were offered testing. This limits the generalisability of the findings presented here.

Fourth, throughout the study period, testing was only offered to individuals who met the epidemiological criteria, were symptomatic, and whose symptoms were among those specified in the case definition in use at the time their symptoms presented. This means symptomatic cases may not have been offered testing because their symptoms were not in the case definition. Given UK testing policy, asymptomatic individuals^1^ were also unlikely to be tested and therefore asymptomatic cases were less likely to be detected, although three cases in the enhanced surveillance and contact tracing dataset were tested despite not reporting any symptoms.

The potential impact of this is two-fold. Misclassification of cases means total case numbers are underestimated and biased towards identifying cases with more severe disease, and estimates of attack rates and the risk of infection in different categories of contacts will also be biased, although in the latter case this non-differential misclassification of SARS-CoV-2 positivity may be less likely to affect the estimate of the relative risk of infection when comparing different categories of contacts.

Fifth, contacts of cases identified towards the end of the study period had less time to develop symptoms and be tested than contacts identified earlier in the study period. The last case for which contact tracing data were collected was reported to PHA on 12 March 2020, meaning their contacts had at most two days to both develop symptoms and get tested. Although it was possible to identify five secondary cases that were hospitalised through linking contact tracing and virology data, any contacts that developed symptoms but did not require hospitalisation were not tested and therefore were not identified. This is again likely to result in underestimation of case numbers and attack rates in different categories of contacts, and may influence the estimates of the relative risk of infection when comparing different categories of contacts.

Sixth, it was not possible to determine how long cases were hospitalised for from the data available. Household contacts of hospitalised cases may potentially be at reduced risk of infection due to reduced exposure to the primary case following the primary case's hospitalisation, with the level of reduction of exposure dependent on the date and duration of hospitalisation. As we were not able to determine and adjust for this in our analysis, our estimate of the SAR among household contacts may be biased, with the assumption being that this bias may result in an underestimate of the SAR among household contacts.

## Discussion

Cumulative incidence of COVID-19 in NI appears to be lower than the rest of the UK, and this may be due to one or more of several factors. NI's physical separation from the UK mainland may have resulted in relatively fewer importations and ‘seeding’ of SARS-CoV-2 cases before control measures were introduced. The first confirmed case in NI was also reported on 27 February 2020, nearly a month after the first case in England on 31 January, which may also explain the lower incidence in NI compared to the rest of the UK. The ROI, with which NI shares an open border, policy of active case finding and testing throughout their epidemic may also have limited the number of potential cross-border importations of COVID-19. Phylogenetic analysis of early samples may enable better understanding of transmission lineages [19] and shed light on the role this may have played in the scale and progress of NI's COVID-19 epidemic. Given the different approaches to testing and surveillance between NI and the ROI, observed differences in cumulative incidence are difficult to interpret.

The data presented here also demonstrate the rapid transition to community transmission of SARS-CoV-2 following the first imported case in NI, and the mechanisms by which this transition may have taken place. It took just 11 days from the first laboratory-confirmed case in NI to the detection of the first sporadic case with no travel history or known links to confirmed cases. In our study we found that household contacts of individuals with laboratory-confirmed SARS-CoV-2 infection are at higher risk of both SARS-CoV-2 infection and symptomatic COVID-19 than high- and low-risk contacts. This finding adds to a growing body of evidence that households are an important locus of community transmission of SARS-CoV-2 [18–22]. Our estimate of the SAR among household contacts was 15.9% (95% CI 6.6%–30.1%), which is similar to those reported in other studies, although higher SARs in household settings have been reported [25, 27]. This may be influenced by differences in testing policy, and/or the number and demographics of individuals occupying the same household, which may affect disease transmission and the susceptibility of individuals to infection. The average number of household contacts per primary case in our study was 1.6 (range: 0–5), which is lower than those reported in studies from China [18, 28] and South Korea [26]. Lower SAR in households with fewer than six members compared to householders with six or more members were also reported in a study from Guanzhou, China [23]. We did not find evidence of variation in the SAR by age group as reported by other studies [6, 18, 23] and this is likely due to the small numbers in our study.

We found that, after adjusting for age and sex of contacts, the odds of testing positive for SARS-CoV-2 was 11.0 (95% CI 1.7–70.3) times higher among household contacts of cases compared to non-household contacts, and this is within the range of estimates from a study in Hangzhou, China [27].

Despite the limitations of our study, this result combined with our estimate of the SAR among household contacts, suggests that control measures aimed at rapidly identifying, testing and isolating household contacts of cases regardless of whether or not they are symptomatic, and reducing the risk of infection in the household, may be effective in preventing transmission of SARS-CoV-2. Rapid identification and testing of household contacts takes on added importance given that we found evidence of possible pre-symptomatic transmission in our study.

As it will still be some time before a vaccine or vaccines are widely available, further research into the effectiveness of non-pharmaceutical interventions (NPIs) in preventing SARS-CoV-2 transmission in the UK is urgently needed to strengthen the public health response to the pandemic, and identify effective interventions to reduce SARS-CoV-2 transmission in the household. One such intervention that may be effective is the use of face coverings and masks. Several recent studies have generated evidence on the effectiveness of face coverings and masks at reducing the risk of SARS-CoV-2 infection generally [27, 29–33], and at reducing transmission within households. Results from a study in China [28] on face mask use among household contacts of individuals with laboratory-confirmed SARS-CoV-2 infection suggest that one or more household members (including the primary case) wearing a face mask at home prior to symptom onset in the primary case was 79% (95% CI 21%–94%) effective at reducing transmission in the home, although it should be noted that data on mask use by study participants was collected via self-reporting rather than direct observation by the study authors. As the authors of this study highlight, the use of face masks have been shown to be effective in reducing transmission of other respiratory viruses [21], including within households under randomised-control trial conditions [34]. Another study from China [25] reported that households that reported not adopting protective measures (defined as wearing masks when contact with index case, hand hygiene after contact with index case and avoiding contact with the index case) after illness onset in the index case had SARs 4.95 (95% CI 1.59–15.39) times higher than households that did report adopting these measures.

Other interventions to prevent transmission within households should also be investigated, and the household setting may present additional opportunities for outbreak control that are not feasible in other areas with increased risk of SARS-CoV-2 transmission such as healthcare facilities, care homes and other institutional settings, especially during periods of increased transmission. A study on household transmission in China reported no secondary SARS-CoV-2 infections among household contacts of cases who self-quarantined at home at the time of symptom onset [18]. Another study from China also reported associations between reduced contact between household members and lower risk of secondary SARS-CoV-2 infection within the household [28]. The ability of individuals to self-quarantine effectively at home is only possible, however, if the household is uncrowded and has sufficient space for infected individuals to minimise contact with other household residents [25, 28]. Future research on reducing household transmission of SARS-CoV-2 could take this further and investigate the effectiveness, feasibility and acceptability of quarantining individuals outside of the home if their household conditions are assessed to be unsuitable for self-quarantine.

Identifying and testing potential interventions takes on added importance given anticipated increased levels of COVID-19 and seasonal increases in other diseases during the coming winter. Preliminary research suggests compliance with ‘lockdown’ measures in the UK has been high [35] despite the many and varied challenges these restrictions on everyday life have imposed, so there may be cause to be optimistic that uptake of interventions aimed at reducing transmission within households may be high if they are tailored to a range of audiences, communicated clearly and implemented effectively [36].

## Data Availability

The cumulative incidence and mortality data that support the findings of this study are openly available through the COVID-19 data dashboards published by the Northern Ireland Department of Health, Public Health England, Public Health Wales, The Government of Scotland and The Government of Ireland. The enhanced surveillance data and contact tracing data used in the study cannot be made available due to data protection requirements.

## Footnotes

1 Testing may have been arranged at the discretion of their primary care provider, or the individuals may have reported symptoms to their primary care provider which they did not later report to PHA surveillance staff.

## Supplementary material

For supplementary material accompanying this paper visit https://doi.org/10.1017/S0950268821000224. click here to view supplementary material
