# Supplementary material for: Epidemiology of COVID-19 in Northern Ireland, 26 February 2020–26 April 2020
Source: Epidemiol Infect. 2021 Jan 29;149:e36. doi: 10.1017/S0950268821000224 (PMC7873460; doi:10.1017/S0950268821000224)
Supplement: Supplementary file 1 [file S0950268821000224sup001.zip › c19_study_appendix_5.docx]

**Epidemiology of COVID-19 in Northern Ireland, 26 February 2020 – 26 April 2020**

**Authors: J. PETT, P. MCALEAVEY, P. MCGURNAGHAN, R. SPIERS, M. O’DOHERTY, L PATTERSON, J. JOHNSTON**

**Appendix 5 – Description of clusters**

The primary cases in Cluster A and Cluster B were both linked to a known case in the ROI, and had travelled as part of a group to Northern Italy. The primary case in Cluster A returned to NI on 29 February 2020 and reported onset of a cough, fever and shortness of breath on the same day. Between 29 February 2020 and 04 March 2020 their partner and three friends, all of whom were categorised as high-risk contacts, also became infected and subsequently tested positive for SARS-COV-2. The primary case’s partner reported headache and fatigue, one of the case’s friends reported chest pain and shortness of breath, and the two other cases in the cluster reported no symptoms.

The primary case in Cluster B, who returned to NI on the same date as the primary case in Cluster A, reported onset of cough and fever on 29 February 2020. Both of the primary case’s household contacts (their partner and child) became infected between 29 February 2020 and 06 March 2020, and developed a cough and fever, with the partner also reporting headache and coryzal symptoms, and the child also reporting sore throat, myalgia, and fatigue.

The primary case in Cluster C was one of the friends of the primary case in Cluster A. Between 02 March 2020 and 06 March 2020 their partner and a relative, household and high risk contacts respectively, became infected. Their partner developed a cough, fever, and shortness of breath, and their relative reported a cough only.

The primary case in Cluster D was linked to a known cluster of COVID-19 cases in the UK, and reported a cough and fever with symptom onset on 01 March 2020, They returned to NI on 28 February 2020, and between 28 February 2020 and 01 March 2020 their partner (a household contact) and a friend they went for dinner with (a high risk contact) on 29 February 2020 became infected. The primary case’s friend reported coryzal symptoms with symptom onset on 04 March 2020, while the partner reported cough, fever, sore throat and chest tightness with symptom onset on 10 March 2020.

The primary case in Cluster E reported cough and fever with symptom onset on 05 March 2020. They had a recent travel history to Italy, and had returned to NI on 02 March 2020. Between 02 March 2020 and 06 March 2020 the primary case’s adult son (a household contact) and a colleague (high risk contact) became infected, with the son reporting cough and fever with symptom onset on 08 March 2020, and the colleague reporting chest pain with symptom onset on 18 March 2020.

The primary case in Cluster F returned from Italy on 04 March 2020 and reported cough and fever with symptom onset on 05 March 2020. Between 04 March 2020 and 11 March 2020 the primary case’s parent, a household contact, became infected and reported a cough with symptom onset on 13 March 2020.
