# Supplementary material for: Epidemiology of COVID-19 in Northern Ireland, 26 February 2020–26 April 2020
Source: Epidemiol Infect. 2021 Jan 29;149:e36. doi: 10.1017/S0950268821000224 (PMC7873460; doi:10.1017/S0950268821000224)
Supplement: Supplementary file 1 [file S0950268821000224sup001.zip › c19_study_appendix_4.docx]

**Epidemiology of COVID-19 in Northern Ireland, 26 February 2020 – 26 April 2020**

**Authors: J. PETT, P. MCALEAVEY, P. MCGURNAGHAN, R. SPIERS, M. O’DOHERTY, L PATTERSON, J. JOHNSTON**

**Appendix 4 – List of data fields collected for enhanced surveillance and contact tracing**

**Enhanced surveillance data**

- **ConCaseNum –** Unique ID of primary case if record is for a secondary case
- **HPZoneCase** – Unique ID within HPZone system
- **PHA_CONTACT_ID** – Unique ID for PHA internal database
- **CurrentStatus** – Alive/Deceased
- **Organisation** – Reporting agency/organisation
- **DateReported** – Date of report
- **DateOfInterview** – Date interview conducted with case
- **NHSNum** – Health and Social care number of case
- **Forename**
- **Surname**
- **Sex**
- **DOB**
- **Age**
- **Postcode**
- **Occupation**
- **HCWTitle** – If a healthcare worker, job title
- **HCWPlace** – If a healthcare worker, place of work
- **DateOnset** – date of reported symptom onset
- **MaxTemp** – maximum temperature recorded up to date of report
- **Fever** – whether case reported having a fever (Yes/No)
- **FeverDateOnset** – Reported date of fever onset
- **Cough** – whether case reported having a cough (Yes/No)
- **CoughDateOnset** – Reported date of cough onset
- **CoughDryProductive** – Whether reported cough was dry or productive
- **ShortnessBreath** – whether case reported experiencing shortness of breath (Yes/No)
- **ShortnessBreathDateOnset** – Reported date of onset of shortness of breath
- **ContactSympCase** – Reported contact with symptomatic case in past 14 days (Yes/No)
- **Management** – whether case is being managed in community or in hospital
- **Admitted** **to hospital** – whether case was hospitalised at time of reporting (Yes/No)
- **HospitalDate1** – Date of hospitalisation
- **SpecDate1** – specimen date
- **LabTestDate1** – date of laboratory testing of specimen

**Contacts data**

**HPZoneCase** – Unique ID in HPZone for primary case

**PHA**_**CONTACT**_**ID** – Unique ID for PHA internal database

**NameCase** – Forename and Surname of primary case

**DateReported** – Date reported to PHA

**NHSNum** – Health and Social Care number of contact

**Forename**

**Surname**

**Sex**

**DOB**

**Age**

**HPZoneContact** – Unique ID in HPZone for contact

**ContactPhone** – Home phone number of contact

**ContactMobile** – Mobile number of contact

**Completed** **follow up** – Whether contact completed 7 or 14 day monitoring period following last reported contact with primary case (Yes/No)

**ID** – Unique ID identifying record within contacts database

**DateLastContactConfirmedCase** – Date of last reported contact with confirmed case

**HCWDateOnsetConfirmedCase** – If contact is a healthcare worker, date of symptom onset in confirmed case

**Contact category** – whether reported contact with primary case was household, high, or low-risk
