# Supplementary material for: Epidemiology of COVID-19 in Northern Ireland, 26 February 2020–26 April 2020
Source: Epidemiol Infect. 2021 Jan 29;149:e36. doi: 10.1017/S0950268821000224 (PMC7873460; doi:10.1017/S0950268821000224)
Supplement: Supplementary file 1 [file S0950268821000224sup001.zip › c19_study_appendix_3.docx]

**Epidemiology of COVID-19 in Northern Ireland, 26 February 2020 – 26 April 2020**

**Authors: J. PETT, P. MCALEAVEY, P. MCGURNAGHAN, R. SPIERS, M. O’DOHERTY, L PATTERSON, J. JOHNSTON**

**Appendix 3 – Categories of key workers eligible for SARS-CoV-2 testing from 4^th^ April 2020 onwards**

- NHS
- Non-NHS healthcare
- Social care
- Medical supply and distribution
- Education and childcare
- Prison and judiciary system
- Funeral industry
- Local and national government
- Food production and distribution
- Defence
- Police
- Fire service
- Transport
- Utilities, communications, broadcasting and financial services
- Manufacturing
- Retail
- Volunteers
- Family members of key workers
