# Supplementary material for: Epidemiology of COVID-19 in Northern Ireland, 26 February 2020–26 April 2020
Source: Epidemiol Infect. 2021 Jan 29;149:e36. doi: 10.1017/S0950268821000224 (PMC7873460; doi:10.1017/S0950268821000224)
Supplement: Supplementary file 1 [file S0950268821000224sup001.zip › c19_study_appendix_2.docx]

**Epidemiology of COVID-19 in Northern Ireland, 26 February 2020 – 26 April 2020**

**Authors: J. PETT, P. MCALEAVEY, P. MCGURNAGHAN, R. SPIERS, M. O’DOHERTY, L PATTERSON, J. JOHNSTON**

**Appendix 2 – Contact category definitions**

| **Category** | **Description** |
| --- | --- |
| **Household** | Household contact- living or spending significant time in the same household  *e.g. living and sleeping in the same home, students in university accommodation sharing kitchen or bathroom facilities, sexual partners*  AND cleaning household-type settings used by the case |
| **High risk** | Persons in healthcare settings (e.g. healthcare workers, cleaners, visitors) who **have not** worn recommended PPE  OR  laboratory workers who **have not** used appropriate laboratory precautions*,  during the following exposures to the patient:  Direct contact with the case or their body fluids or their laboratory specimens  OR  presence in the same room of a healthcare setting when an aerosol generating procedure is undertaken on the case |
| **Low risk** | Persons in healthcare settings (e.g. healthcare workers, cleaners) who have worn recommended PPE during all the following exposures to the patient:  Direct contact with the case or their body fluids or their laboratory specimens,  OR  presence in the same room of a healthcare setting when an aerosol generating procedure is undertaken on the case |
| **High risk** | **For any other (non-aircraft) exposure not satisfying in categories A-C:**  Direct contact or face to face contact with case e.g. talking, being coughed on **for** any length of time  *NB: this can include healthcare settings and community settings if exposures do not meet the above categories* |
| **Low risk** | **For any other (non-aircraft) exposure not satisfying categories A-D:**  being ≤2 metres of the case for >15 minutes.  *NB: this can include healthcare settings and community settings if exposures do not meet the above categories* |
| **Low risk** | **Aircraft-specific:**  Passengers in 2 seats in all directions around the case  AND  Cabin crew serving the area where case seated |
