# Supplementary material for: Epidemiology of COVID-19 in Northern Ireland, 26 February 2020–26 April 2020
Source: Epidemiol Infect. 2021 Jan 29;149:e36. doi: 10.1017/S0950268821000224 (PMC7873460; doi:10.1017/S0950268821000224)
Supplement: Supplementary file 1 [file S0950268821000224sup001.zip › c19_study_appendix_1.docx]

**Epidemiology of COVID-19 in Northern Ireland, 26 February 2020 – 26 April 2020**

**Authors: J. PETT, P. MCALEAVEY, P. MCGURNAGHAN, R. SPIERS, M. O’DOHERTY, L PATTERSON, J. JOHNSTON**

**Appendix 1 – Case definitions**

| **Case definition** | **Date published** | **Case definition – Suspected case** |
| --- | --- | --- |
| **1** | 15 Jan 2020 | Severe acute respiratory infection requiring admission to hospital with clinical or radiological evidence of pneumonia or acute respiratory distress syndrome  OR  Fever, or history of fever and acute respiratory infection (sudden onset of respiratory infection with at least one of: shortness of breath, cough or sore throat)  AND  has travelled to Wuhan City in the 14 days before the onset of illness  OR  has been in contact with confirmed cases of COVID-19 |
| **2** | 31 Jan 2020 | **Geographic boundary changed** to include travel to Mainland China (excluding Taiwan, Hong Kong, Macau)  **Clinical criteria changed** with removal of ‘sore throat’ from clinical criteria |
| **3** | 6 Feb 2020 | **Geographic boundary changed** to include travel to mainland China, Hong Kong, Japan, Macau, Malaysia, Republic of Korea, Singapore, Taiwan, or Thailand in the 14 days prior to the onset of symptoms  **Clinical criteria changed** to include ‘Fever with no other symptoms’ |
| **4** | 25 Feb 2020 | Change to geographic criteria and introduction of higher and lower risk categories.  **Lower risk** defined as travel to China, Thailand, Japan, Republic of Korea, Hong Kong, Taiwan, Singapore, Malaysia, Macau in the previous 14 days; or  Northern Italy (not including, Pisa, Florence and Rimini), Vietnam, Cambodia, Laos, Myanmar since February 19th 2020  **Higher risk** defined as travel to Hubei Province (including Wuhan) in the previous 14 days, or travel to Iran, specific lockdown areas in Northern Italy, or special care zones of South Korea from February 19^th^, 2020 |
| **5** | 13 Mar 2020 | **Geographical boundary and clinical criteria changed**  Travel removed, with all symptomatic individuals considered as ‘possible’ cases, defined as new continuous cough and  **Inpatient definition added**  requiring admission to hospital (a hospital practitioner has decided that admission to hospital is required with an expectation that the patient will need to stay at least one night)  **and** have either clinical or radiological evidence of pneumonia  **or** acute respiratory distress syndrome  **or** influenza like illness (fever ≥37.8°C and at least one of the following respiratory symptoms, which must be of acute onset: persistent cough (with or without sputum), hoarseness, nasal discharge or congestion, shortness of breath, sore throat, wheezing, sneezing. |
